# Effect of γ-lactones and γ-lactams compounds on *Streptococcus mutans* biofilms

**DOI:** 10.1590/1678-7757-2017-0065

**Published:** 2018-02-15

**Authors:** Mariane Beatriz Sordi, Thaís Altoé Moreira, Juan Felipe Dumes Montero, Luis Cláudio Barbosa, César Augusto Magalhães Benfatti, Ricardo de Souza Magini, Andréa de Lima Pimenta, Júlio César Matias de Souza

**Affiliations:** 1 Universidade Federal de Santa Catarina Universidade Federal de Santa Catarina Departamento de Odontologia Centro de Ensino e Pesquisa em Implantes Dentários FlorianópolisSanta Catarina Brasil Universidade Federal de Santa Catarina, Departamento de Odontologia, Centro de Ensino e Pesquisa em Implantes Dentários (CEPID), Florianópolis, Santa Catarina, Brasil.; 2 Universidade Federal de Minas Gerais Universidade Federal de Minas Gerais Instituto de Ciências Exatas Departamento de Química Belo HorizonteMinas Gerais Brasil Universidade Federal de Minas Gerais, Instituto de Ciências Exatas, Departamento de Química, Belo Horizonte, Minas Gerais, Brasil.; 3 Universidade Federal de Santa Catarina Universidade Federal de Santa Catarina Departamento de Engenharia Química Laboratório de Tecnologias Integradas FlorianópolisSanta Catarina Brasil Universidade Federal de Santa Catarina, Departamento de Engenharia Química, Laboratório de Tecnologias Integradas (InteLAB), Florianópolis, Santa Catarina, Brasil.; 4 Université de Cergy-Pontoise Université de Cergy-Pontoise Département de Biologie France Université de Cergy-Pontoise, Département de Biologie, Cergy-Pontoise, France.

**Keywords:** Lactones, Lactams, Biofilm, Antimicrobial, Streptococcus mutans

## Abstract

Considering oral diseases, antibiofilm compounds can decrease the accumulation of pathogenic species such as *Streptococcus mutans* at micro-areas of teeth, dental restorations or implant-supported prostheses.

**Objective:**

To assess the effect of thirteen different novel lactam-based compounds on the inhibition of *S. mutans* biofilm formation.

**Material and methods:**

We synthesized compounds based on γ-lactones analogues from rubrolides by a mucochloric acid process and converted them into their corresponding γ-hydroxy-γ-lactams by a reaction with isobutylamine and propylamine. Compounds concentrations ranging from 0.17 up to 87.5 μg mL-1 were tested against *S. mutans*. We diluted the exponential cultures in TSB and incubated them (37°C) in the presence of different γ-lactones or γ-lactams dilutions. Afterwards, we measured the planktonic growth by optical density at 630 nm and therefore assessed the biofilm density by the crystal violet staining method.

**Results:**

Twelve compounds were active against biofilm formation, showing no effect on bacterial viability. Only one compound was inactive against both planktonic and biofilm growth. The highest biofilm inhibition (inhibition rate above 60%) was obtained for two compounds while three other compounds revealed an inhibition rate above 40%.

**Conclusions:**

Twelve of the thirteen compounds revealed effective inhibition of *S. mutans* biofilm formation, with eight of them showing a specific antibiofilm effect.

## Introduction

Biofilm consists in a complex microbial community embedded in an exopolymeric matrix based on polysaccharides, glycoproteins, nucleic acids and water^13^^,^^26^. Biofilms are found in nature adhered to different surfaces depending on nutritional and environmental conditions, such as oxygen, pH and nutrients. Factors related to the surface itself, like chemical composition, surface energy and roughness, also affect biofilm formation and development^2^^,^^13^. In oral environment, biofilm accumulation by pathogenic microorganisms is associated to oral diseases, such as gingivitis, periodontitis, peri-implantitis and caries^13^^,^^22^. Over the last decades, conventional therapy on the use of antimicrobial agents targeting bacterial cell viability has significantly decreased the impact of infectious diseases. Nevertheless, such achievement has stimulated the development of microbial resistance to antibiotics^1^^,^^4^^,^^5^^,^^20^.

The development of new antimicrobial compounds targeting bacterial virulence instead of bacterial viability is a promising strategy for the prevention and treatment of infectious diseases, avoiding the stimulation of microbial resistance. In addition, novel anti-virulence drugs should disturb the adherence of pathogenic species to biotic and abiotic surfaces, eliminating the infection by the host immune system. Furthermore, virulence-targeted drugs can be associated with conventional antibiotics to improve their effectiveness^1^^,^^4^^,^^5^^,^^20^.

Considering oral diseases, antibiofilm compounds can decrease the presence of pathogenic species such *Streptococcus mutans* on exposed and retentive micro-areas of teeth, dental restorations or implant-supported prostheses^10^^,^^21^. For adhesion to oral surfaces, *S. mutans* produces extracellular polysaccharides that protect the consortia of bacteria against antimicrobial substances (*e.g.* antibiotics)^3^^,^^13^^,^^23^. Biofilms involving significant *S. mutans* concentration result in a release of high content of lactic acid that decreases the local pH, leading to the demineralization of tooth tissues and restorative structural materials^13^^,^^21^.

Studies on the formation and inhibition of biofilms discovered bacterial-communication systems – namely, quorum sensing (QS), which orchestrate important temporal events during the infection process^6^^,^^7^^,^^24^. Natural quorum sensing inhibitors (QSIs), such as halogenated furanones derivatives, have revealed inhibitory activity on the expression of several virulence factors in *Pseudomonas aeruginosa* biofilms, increasing the sensitivity of the biofilm to conventional antibiotics^8^^,^^9^. On oral *Streptococci*, brominated furanones were able to inhibit biofilm formation without affecting planktonic growth^12^. That is an important feature to avoid in the future development of drug resistance. However, previous studies have reported a high chemical reactivity and cytotoxicity of furanones and some of their derivatives^8^^,^^9^, which led to the development of similar synthetic biocompatible compounds, such as γ-lactones and their derived γ-lactams. Different lactam-based compounds have demonstrated potential effect on biofilm inhibition, providing an alternative therapeutic approach for the treatment of biofilm-related chronic infectious diseases^11^^,^^15^^,^^16^.

Previous studies revealed that synthetic lactams and lactones, similar to natural rubrolides, were mainly active against *Enterococcus faecalis* biofilms^16^. On another study, a group of 28 lactones and lactams were tested against different biofilms produced by *Staphylococcus aureus*, *Pseudomonas aeruginosa*, *Staphylococcus epidermidis* and *Streptococcus mutans*. Several compounds showed biofilm inhibition activity against all the species mentioned, particularly on *S. epidermidis* and *P. aeruginosa*^15^. Such results encourage the development of further experiments to identify other lactones and lactams as potential antibiofilm agents to be used in the control of infections by oral pathogens.

Thus, the aim of this study was to assess the effect of thirteen novel synthetic compounds based on γ-lactones or γ-lactams on the inhibition of *Streptococcus mutans* biofilms. We hypothesized that γ-lactones and γ-lactams could inhibit the *S. mutans* biofilm formation without interfering in microbial growth, avoiding the development of bacterial resistance.

## Material and methods

### Bacterial strains and growth conditions

*S. mutans* ATCC 25175 were microaerophilically grown for 48 h at 37°C in agar plates with 32 g/L of BHI agar (Bacto, Difco, Radnor, Pennsylvania, USA). Bacterial cells were inoculated in Tryptic Soy Broth (TSB, Bacto, Difco, Radnor, Pennsylvania, USA) supplemented with 200 g/L sucrose for 24 h at 37°C and 150 rpm. After incubation, cells were harvested by centrifugation for 10 min at 4°C and 5000 rpm and washed twice with Phosphate Buffer Solution (PBS, Sigma-Aldrich, St. Louis, Missouri, USA). Then, cells were re-suspended in TSB supplemented with 200 g/L sucrose to obtain an optical density (OD) of about 0.6 at Abs_630_, corresponding to approximately 1×10^8^ CFU/mL. We measured the OD at 630 nm using a spectrophotometer (Tecan Infinite M200, Meilen, Zurich, Switzerland)^15^^,^^16^^,^^18^^,^^19^.

### Synthesis of antibiofilm compounds

**Figure 1:**
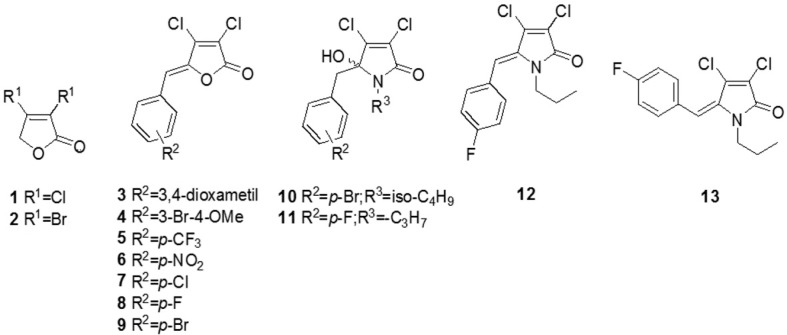
General structure for the test rubrolides-based γ-lactones compounds

We synthesized thirteen different anti-biofilm compounds based on γ-lactones and γ-lactams for biofilm assays. Afterwards, we synthesized compounds formulated with rubrolides-based γ-lactones by mucochloric acid treatment (Figure 1). Then, we converted γ-lactones into their corresponding γ-hydroxy-γ-lactams by a reaction with isobutylamine and propylamine, as described by Pereira, et al.^15^ (2014). A regioselective Suzuki-Miyaura cross-coupling between lactones and arylboronic acids in the presence of Ag_2_O, AsPh_3_ and catalytic amount of PdCl_2_(MeCN)_2_ resulted in the formation of 4-aryl-3-bromofuran-2(5H)-ones in low yields (26-37%). The low conversions of these Suzuki cross-couplings reactions can be explained in part by the formation of the biphenyls 2,20-dimethoxybiphenyl, 5,50-dibromo-2,20-dimethoxybiphenyl, 5,50-dimethyl-2,20-dimethoxybiphenyl, and 5,50-dichloro-2,20-dimethoxybiphenyl produced in 14-23% as a result of the homocoupling of the boronic acids^17^. On the next step, we treated 4-aryl-3-bromofuran-2(5H)-ones with aromatic aldehyde, tert-butyldimethylsilyl trifluoromethanesulfonate (TBDMSOTf), diisopropylethylamine (DIPEA) followed by treatment of the silyl ether generated *in situ* with DBU. A subsequent treatment of the resultant compounds with an excess of isobutylamine resulted in the formation of the corresponding g-hydroxy-glactams in good yields (76-85%). All spectroscopic and physical characterization for the compounds were in agreement with the values reported on supplementary information described in previous studies^15^^–^^17^.

### Microbiological assays

All compounds were solubilized in dimethyl sulfoxide (DMSO) at 5 mg/mL^-1^. For the microbiological assays, that stock solution was further diluted to concentrations ranging from 0.17 up to 87.5 μg/mL^-1^. The DMSO concentration was standardized at 3.5% in all microbiological assays, to avoid side effects on bacterial growth. Previous results indicated that 3.5% DMSO is innocuous both to bacterial growth and biofilm formation^15^^,^^16^^,^^18^^,^^19^.

Biofilm formation assays were performed in 96-well plates, each containing 100 μL of *S. mutans* inoculum at 5×10^8^ CFU/mL in TSB supplemented with 20% sucrose (w/v) and 3.5% DMSO (v/v) to ensure that the concentration of this solvent remained constant as the tested molecules were sequentially diluted for the inhibition assays. For each assay, we added 100 μL of the tested compound (175 μg/mL^-1^ in 3.5% DMSO) to the first well and serially diluted it at a 1:2 ratio by transferring 100 μL of its content to the next well, down to the final concentration at 0.17 μg/mL. Control groups contained all the mentioned components in the absence of the inhibitor compound (positive control) or in the absence of bacteria (negative control). Microplates were incubated at 37°C in a humidified chamber for 24 h. Each assay was performed in triplicate^15^^,^^16^^,^^18^^,^^19^.

To assess effects on bacterial growth prior to biofilm analyses, we quantified bacterial growth by absorbance readings (630 nm) using a microtiter plate reader device (Tecan Infinite M200, Meilen, Zurich, Switzerland). For biofilm analyses by the crystal violet (CV) staining method, we discarded bacterial suspensions from the 96-well plates and washed the wells three times with distilled water. In each well, we added 120-μl CV solution (0.1% w/v) and allowed it to stain the adherent biofilm for 15 min. We removed the CV solution and washed the wells three times with distilled water. Afterwards, we added 120 μl of sodium dodecyl sulfate at 1% (w/v) to solubilize the CV and determine the optical density of biofilms by spectrophotometry at 595 nm (Tecan Infinite M200, Meilen, Zurich, Switzerland)^15^^,^^16^^,^^18^^,^^19^.

### Statistical analysis

We statistically analyzed the data by one-way ANOVA using the STATISTICA 6.0^®^ software (StatSoft, Palo Alto, California, USA). The level of statistical significance was set at 0.05. We performed the T-test for independent samples to discover which lactams significantly affected the inhibition of the *S. mutans* biofilm.

## Results

**Figure 2:**
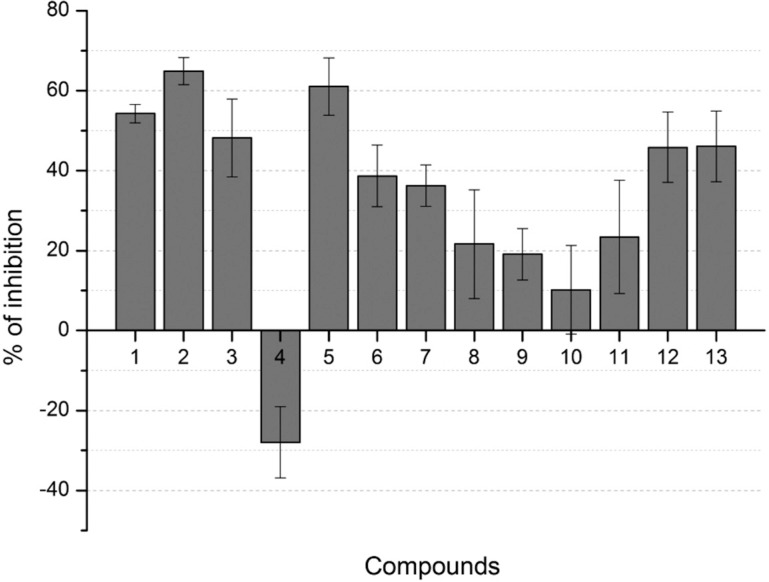
Antibiofilm activity of compounds 1-13, at their most effective concentrations for *S. mutans* biofilm inhibition. The percent inhibition plot data were calculated by taking the measures of the positive control as 100% of biofilm formation

Results obtained for inhibition of planktonic growth and biofilm formation of *S. mutans* are shown in Figure 2. For each compound tested, values for inhibition of planktonic growth and biofilm were indicated as percentages of biofilm produced by the positive control, at the concentrations that each compound showed the highest biofilm inhibitory effect.

Biofilm analyses showed that twelve of the thirteen tested compounds were active for inhibition of *S. mutans* biofilm formation. Compound 4 induced biofilm formation (at 87.5 μg/mL^-1^). That was previously reported for other rubrolide analogues^15^. Regarding planktonic growth, only compound 8 (at 0.34 μg/mL^-1^) revealed a mild inhibitory effect (19%), while several compounds were able to induce planktonic growth, as illustrated for compound 11 that caused 35.8% growth induction (at 43.8 μg/mL^-1^). For a third group, represented by compounds 2-6, the effect on the planktonic growth was negligible.

**Figure 3:**
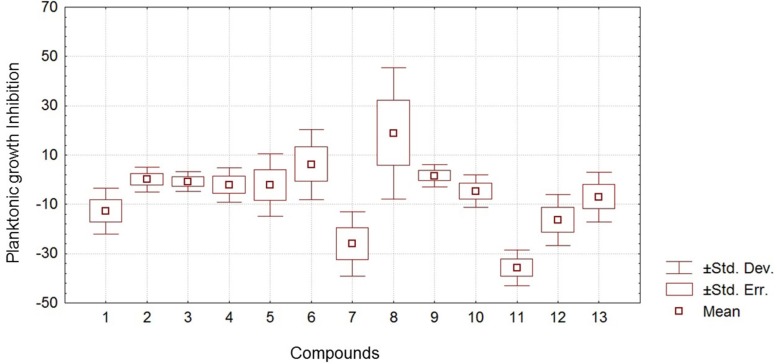
Statistical results for each compound on inhibition of planktonic growth obtained by ANOVA

**Figure 4:**
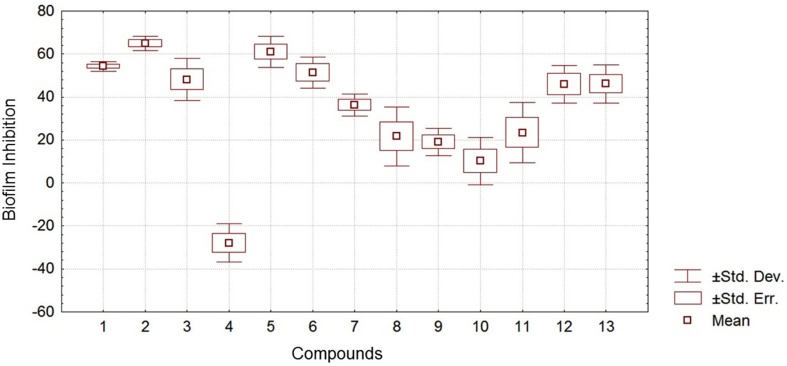
Statistical results for each compound on inhibition of biofilm formation obtained by ANOVA

**Figure 5:**
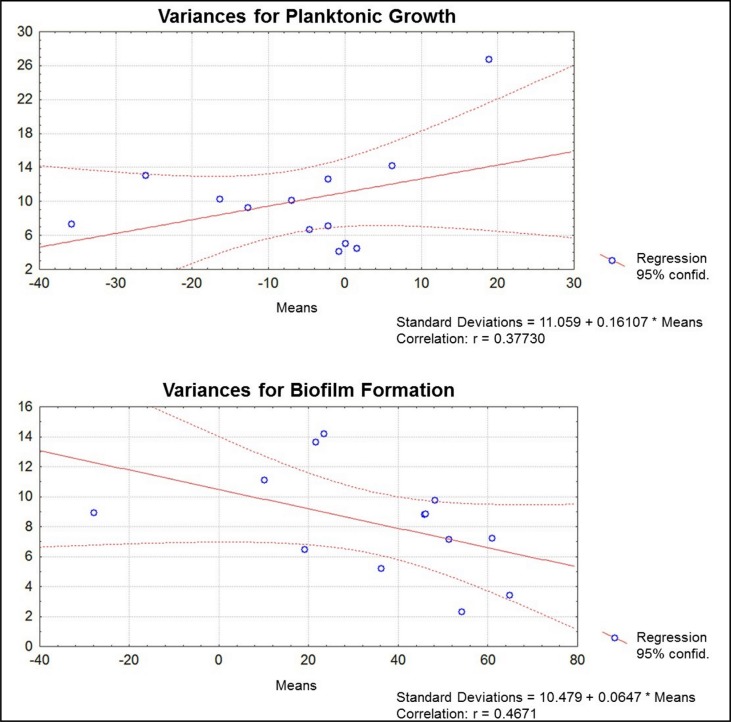
Analysis of variance of the results

As shown in Figure 2, compounds 1-5, 12, and 13 caused more than 40% biofilm inhibition, at different concentration for each compound. Clearly, the most active compound was the simple lactone 2, with a biofilm inhibition rate of 65% at 0.17 μg/mL^-1^. The chlorine analogue 1 was less active, causing 54% biofilm inhibition at 5.44 μg/mL^-1^. Statistical analysis confirmed the significance of the results (Figures 3-5).

**Figure 6:**
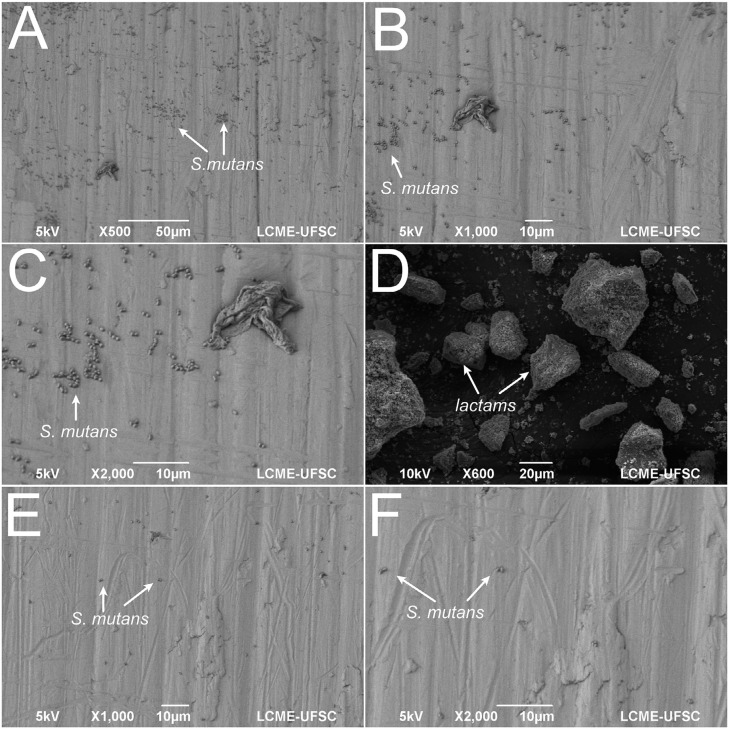
Scanning electron microscopy images of (A-C) *Streptococcus mutans* free of lactam effect, (D) lactam powder particle and (E-F) *S. mutans* in the presence of lactam

In Figure 6, scanning electron microscopy images are shown for *Streptococcus mutans* free of lactam effect, lactam powder particle and *S. mutans* in the presence of lactam.

## Discussion

In this study, we evaluated the effects of thirteen different compounds based on γ-lactones and γ-lactams against *S. mutans* biofilm formation, since some structurally similar lactones and lactams had already been previously classified as potential antibiofilm agents against other bacterial species^15^^,^^16^. Twelve of the thirteen tested compounds were effective in inhibiting the *S. mutans* biofilm formation. Eight of the thirteen compounds inhibited *S. mutans* biofilm formation without interfering with bacterial viability, as measured by the planktonic growth. Such results confirmed the hypothesis proposed in this study on the potential of γ-lactones and γ-lactams for the development of a new generation of antibacterial drugs, targeting bacterial virulence instead of viability. Since several tested compounds did not show inhibition of bacterial growth while inhibiting biofilm formation, it is predicted that these γ-lactones and γ-lactams are specifically active against biofilm and less prone to induce the development of bacterial resistance.

In a very similar study^15^, a group of 28 compounds – including brominated furanones and their corresponding lactams – were assessed for their antibiofilm activity. The lactam-based compounds were active against biofilm produced by Gram-positive and Gram-negative bacteria. Considering *S. mutans*, nineteen compounds were able to inhibit biofilm formation; however, eleven of those revealed an inhibition percentage below 15%. In our study, the results revealed that twelve of the thirteen compounds tested were able to inhibit from 65% to 5.6% of biofilm formation (Figure 2). The most active lactam compounds found in the previous study revealed biofilm inhibition percentages at 89, 34, and 31.7%, respectively, while this study revealed biofilm inhibition percentages at 65, 61, and 54.3%.

Previous studies showed reduced CFU counting in the biofilm detached from titanium surfaces exposed to lactam-derived compounds (1.5×10^2^ CFU/mL in the presence of lactam and 4×10^2^ CFU/mL in its absence)^26^ (Figure 6). The incorporation of organic antibiofilm compounds based on lactams was successfully carried out in a previous study by functionalizing sPEEK (sulphonated poly-ether-ether-ketone)^14^. The sulphonation treatment is useful to embed therapeutic compounds such as lactam-based antibiofilms. A significant inhibition of biofilm effect was detected on sPEEK surfaces containing lactams and, therefore, no effect was noticed on the physiologic planktonic growth of *S. mutans*. Such results indicated that the incorporation of lactams into materials used for dental rehabilitations, such as titanium or sPEEK, represents an effective clinical evolution regarding novel biomaterials with specific antibiofilm properties^14^. Additionally, further studies could evaluate the incorporation of antibiofilms in other polymers, such as poly(lactic-co-glycolic) acid, or PLGA, to cover titanium dental abutments or even to produce scaffolds for bone regeneration.

The development of biocompatible lactones and lactam-based compounds is an important strategy to avoid stimulating bacterial resistance^1^^,^^2^^,^^4^^,^^5^. Besides, previous studies had shown that lactams are less cytotoxic than furanones^8^^,^^9^ and more reactive against biofilm formation than their corresponding lactones^15^^,^^16^. It is important to notice that results presented here do not preclude that other metabolic pathways, indirectly related to biofilm formation, could also be affected at different concentrations than those at which the tested compounds display antibiofilm activity. The absence of a direct dose-dependence correlation for biofilm inhibition revealed by several compounds tested could be attributed to synergistic or antagonist activities of γ-lactones and γ-lactams at different concentrations over other metabolic pathways, which could indirectly affect biofilm formation or lead to a biofilm inhibition at low concentrations^15^.

## Conclusions

This study assessed the effect of thirteen different compounds based on γ-lactones and γ-lactams against *S. mutans* biofilm formation. Eight of the tested compounds were active against *S. mutans* biofilms without showing a significant interference with bacteria viability, as assessed by planktonic growth measurements. The most active compounds described in this study revealed an inhibition rate at 65% against *S. mutans* biofilm. Thus, compounds tested have great potential to inhibit biofilm formation over different restorative materials and are good candidates for the development of new specific antibiofilm drugs, avoiding the development of bacterial resistance. Nevertheless, further studies should be performed to evaluate their effects against formation of multispecies biofilms.

## References

[1] 1- Barczak AK, Hung DT. Productive steps toward an antimicrobial targeting virulence. Curr Opin Microbiol. 2009;12(5):490-6. 10.1016/j.mib.2009.06.012 PMC2763366 19631578

[2] 2- Boles BR, Thoendel M, Singh PK. Self-generated diversity produces “insurance effects” in biofilm communities. Proc Natl Acad Sci U S A. 2004;101(47):16630-5. 10.1073/pnas.0407460101 PMC528905 15546998

[3] 3- Bowen WH, Koo H. Biology of *Streptococcus mutans*-derived glucosyltransferases: role in extracellular matrix formation of cariogenic biofilms. Caries Res. 2011;45(1):69-86. 10.1159/000324598 PMC3068567 21346355

[4] 4- Cegelski L, Marshall GR, Eldridge GR, Hultgren SJ. The biology and future prospects of antivirulence therapies. Nat Rev Microbiol. 2008;6(1):17-27. 10.1038/nrmicro1818 PMC2211378 18079741

[5] 5- Clatworthy AE, Pierson E, Hung DT. Targeting virulence: a new paradigm for antimicrobial therapy. Nat Chem Biol. 2007;3(9):541-8. 10.1038/nchembio.2007.24 17710100

[6] 6- Costerton JW. Bacterial biofilms: a common cause of persistent infections. Science. 1999;284(5418):1318-22. 10.1126/science.284.5418.1318 10334980

[7] 7- Fuqua WC, Winans SC, Greenberg EP. Quorum sensing in bacteria: the LuxR-LuxI family of cell density responsive transcriptional regulators. J Bacteriol. 1994;176(2):269-75. 10.1128/jb.176.2.269-275.1994 PMC205046 8288518

[8] 8- Hentzer M, Riedel K, Rasmussen TB, Heydorn A, Andersen JB, Parsek MR, et al. Inhibition of quorum sensing in *Pseudomonas aeruginosa* biofilm bacteria by a halogenated furanone compound. Microbiology. 2002;148(Pt 1):87-102. 10.1099/00221287-148-1-87 11782502

[9] 9- Hentzer M, Wu H, Andersen JB, Riedel K, Rasmussen TB, Bagge N, et al. Attenuation of *Pseudomonas aeruginosa* virulence by quorum sensing inhibitors. EMBO J. 2003;22(15):3803-15. 10.1093/emboj/cdg366 PMC169039 12881415

[10] 10- Huang L, Xu QA, Liu C, Fan MW, Li YH. Anti-caries DNA vaccine-induced secretory immunoglobulin A antibodies inhibit formation of *Streptococcus mutans* biofilms *in vitro*. Acta Pharmacol Sin. 2013;34(2):239-46. 10.1038/aps.2012.145 PMC4089030 23274411

[11] 11- Kim SG, Yoon YH, Choi JW, Rha KS, Park YH. Effect of furanone on experimentally induced *Pseudomonas aeruginosa* biofilm formation: *in vitro* study. Int J Pediatr Otorhinolaryngol. 2012;76(11):1575-8. 10.1016/j.ijporl.2012.07.015 22884365

[12] 12- Lonn-Stensrud J, Petersen FC, Benneche T, Scheie AA. Synthetic bromated furanone inhibits autoinducer-2-mediated communication and biofilm formation in oral streptococci. Oral Microbiol Immunol. 2007;22(5):340-6. 10.1111/j.1399-302X.2007.00367.x 17803632

[13] 13- Marsh PD, Martin MV. Oral microbiology. Edinburgh: Elsevier; 2009.

[14] 14- Montero JF, Barbosa LC, Pereira UA, Barra GM, Fredel MC, Benfatti CA, et al. Chemical, microscopic, and microbiological analysis of a functionalized poly-ether-ether-ketone-embedding antibiofilm compounds. J Biomed Mater Res A. 2016;104(12):3015-20. 10.1002/jbm.a.35842 27458927

[15] 15- Pereira UA, Barbosa LC, Maltha CR, Demuner AJ, Masood MA, Pimenta AL. γ-Alkylidene-γ-lactones and isobutylpyrrol-2(5H)-ones analogues to rubrolides as inhibitors of biofilm formation by gram-positive and gram-negative bacteria. Bioorg Medic Chem Lett. 2014;24(4):1052-6. 10.1016/j.bmcl.2014.01.023 24484899

[16] 16- Pereira UA, Barbosa LC, Maltha CR, Demuner AJ, Masood MA, Pimenta AL. Inhibition of *Enterococcus faecalis* biofilm formation by highly active lactones and lactams analogues of rubrolides. Eur J Medic Chem. 2014;82:127-38. 10.1016/j.ejmech.2014.05.035 24880232

[17] 17- Pereira UA, Moreira TA, Barbosa LC, Maltha CR, Bomfim IS, Maranhão SS, et al. Rubrolide analogues and their derived lactams as potential anticancer agents. Med Chem Comm. 2016;7:345-52.

[18] 18- Pimenta AL, Chiaradia-Delatorre LD, Mascarello A, Oliveira KA, Leal PC, Yunes RA, et al. Synthetic organic compounds with potential for bacterial biofilm inhibition, a path for the identification of compounds interfering with quorum sensing. Int J Antimicrob Agents. 2013;42(6):519-23. 10.1016/j.ijantimicag.2013.07.006 24016798

[19] 19- Pimenta AL, Di Martino P, Le Bouder E, Hulen C, Blight MA. *In vitro* identification of two adherence factors required for *in vivo* virulence of *Pseudomonas fluorescens*. Microbes Infect. 2003;5(13):1177-87. 10.1016/j.micinf.2003.09.002 14623013

[20] 20- Rasko DA, Sperandio V. Anti-virulence strategies to combat bacteria-mediated disease. Nat Revs Drug Discov. 2010;9(2):117-28. 10.1038/nrd3013 20081869

[21] 21- Souza JC, Ponthiaux P, Henriques M, Oliveira R, Teughels W, Celis JP, et al. Corrosion behaviour of titanium in the presence of *Streptococcus mutans*. J Dent. 2013;41(6):528-34. 10.1016/j.jdent.2013.03.008 23578470

[22] 22- Teughels W, Van Assche N, Sliepen I, Quirynen M. Effect of material characteristics and/or surface topography on biofilm development. Clin Oral Implants Res. 2006;17:68-81. 10.1111/j.1600-0501.2006.01353.x 16968383

[23] 23- Welin-Neilands J, Svensäter G. Acid tolerance of biofilm cells of *Streptococcus mutans*. 2007;73(17):5633-8. 10.1128/AEM.01049-07 PMC2042095 17630302

[24] 24- Whitehead NA, Barnard AM, Slater H, Simpson NJ, Salmond GP. Quorum-sensing in Gram-negative bacteria. FEMS Microbiol Revs. 2001;25(4):365-404. 10.1111/j.1574-6976.2001.tb00583.x 11524130

[25] 25- Xavier JG, Geremias TC, Montero JF, Vahey BR, Benfatti CA, Souza JC, et al. Lactam inhibiting *Streptococcus mutans* growth on titanium. Mater Sci Eng C Mater Biol App. 2016;68:837-41. 10.1016/j.msec.2016.07.013 27524086

[26] 26- Zijnge V, van Leeuwen MBM, Degener JE, Abbas F, Thurnheer T, Gmur R, et al. Oral biofilm architecture on natural teeth. PloS One. 2010;5(2):e9321. 10.1371/journal.pone.0009321 PMC2827546 20195365

